# CanVar: A resource for sharing germline variation in cancer patients

**DOI:** 10.12688/f1000research.10058.1

**Published:** 2016-12-05

**Authors:** Daniel Chubb, Peter Broderick, Sara E. Dobbins, Richard S. Houlston

**Affiliations:** 1 Division of Genetics and Epidemiology, The Institute of Cancer Research, London, UK; 2 Division of Molecular Pathology, The Institute of Cancer Research, London, UK

**Keywords:** exome sequencing, ExAC, CanVar, cancer, colorectal cancer, NGS, Germline, database

## Abstract

The advent of high-throughput sequencing has accelerated our ability to discover genes predisposing to disease and is transforming clinical genomic sequencing. In both contexts knowledge of the spectrum and frequency of genetic variation in the general population and in disease cohorts is vital to the interpretation of sequencing data. While population level data is becoming increasingly available from publicly accessible sources, as exemplified by The Exome Aggregation Consortium (ExAC), the availability of large-scale disease-specific frequency information is limited. These data are of particular importance to contextualise findings from clinical mutation screens and small gene discovery projects. This is especially true for cancer, which is typified by a number of hereditary predisposition syndromes.  Although mutation frequencies in tumours are available from resources such as Cosmic and The Cancer Genome Atlas, a similar facility for germline variation is lacking. Here we present the Cancer Variation Resource (CanVar) an online database which has been developed using the ExAC framework to provide open access to germline variant frequency data from the sequenced exomes of cancer patients. In its first release, CanVar catalogues the exomes of 1,006 familial early-onset colorectal cancer (CRC) patients sequenced at The Institute of Cancer Research. It is anticipated that CanVar will host data for additional cancers, providing a resource for others studying cancer predisposition and an example of how the research community can utilise the ExAC framework to share sequencing data.

## Introduction

With the widespread adoption of high-throughput sequencing as a tool for disease gene discovery and clinical diagnostics there is a need to evaluate candidate disease predisposition genes through defining the spectrum and frequency of genetic variation in the general population and in specific disease cohorts. For this to be meaningful, large sample sizes are required in order that variant frequencies are accurately defined. Such data is often only acquired through combining multiple datasets. Although these data are being rapidly produced by both large consortia and individual research groups, their acquisition and integration are subject to logistical, computational and ethical challenges. When undertaken by multiple agencies, this results in considerable duplication of effort, the products of which may not be widely shared. It is therefore desirable for large, processed sequencing datasets to be made easily accessible to the community. Recently, a paradigm for sharing has been provided by the Exome Aggregation Consortium ^1, 2^ (ExAC). ExAC have aggregated and analysed a set of 60,706 exomes from over twenty different studies, providing this information as an intuitive online resource. The ExAC website presents these data as variant frequencies stratified by different ethnic groups alongside additional sequencing quality metrics and transcript based annotations.

Similar resources providing frequencies of variants in specific disease associated cohorts are not widely available. Such datasets are of particular importance for small-scale studies, where the confirmation of rare variant frequencies in genes of interest is critical to determine the importance of candidate genes. Furthermore, in the case of clinical genetic testing, they aid in the interpretation of variants of unknown significance. This is especially true for cancer, where it is estimated that 5–10% of cases have a strong heritable basis ^3^. The identification of genes involved in hereditary cancers not only provide valuable biological insight but can allow for screening of at risk individuals, providing an opportunity for early diagnosis, which is key to long-term survival. To address the deficiency of germline frequency data in the realm of cancer research, we have produced CanVar, an online resource derived from cancer patient germline exome sequencing data. CanVar has been produced by adapting the ExAC framework ^2^ to provide cancer type specific variant frequencies, presenting them as a familiar and intuitive online interface modelled after the ExAC browser.

### CanVar datasets

CanVar currently catalogues frequency data for 1,006 early-onset familial colorectal cancer cases ^4^. In total, 1,096,907 variant sites are catalogued in CanVar: specifically 981,491 single nucleotide variants (SNVs) and 115,416 insertion deletions (indels). As previous studies have observed, rare variation is itself common, indeed 52% of these variants are only observed in one sample.

It is beneficial to be able to compare cancer variant frequency in cases with that observed in population frequency control data. We have therefore annotated each cancer variant with ExAC allele frequency data excluding samples from The Cancer Genome Atlas (TCGA, n=53,105, henceforth referred to as non-TCGA ExAC). Links are also provided to the relevant ExAC browser entries at the gene and variant levels in order to assess loss of function tolerance and overall gene burden.

### CanVar website

**Figure 1.**
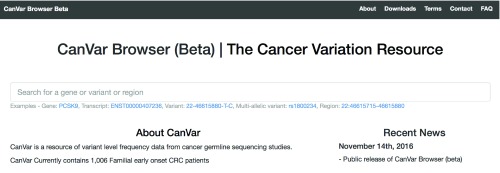
The CanVar front page features a search bar, example queries and additional news and updates.

**Figure 2.**
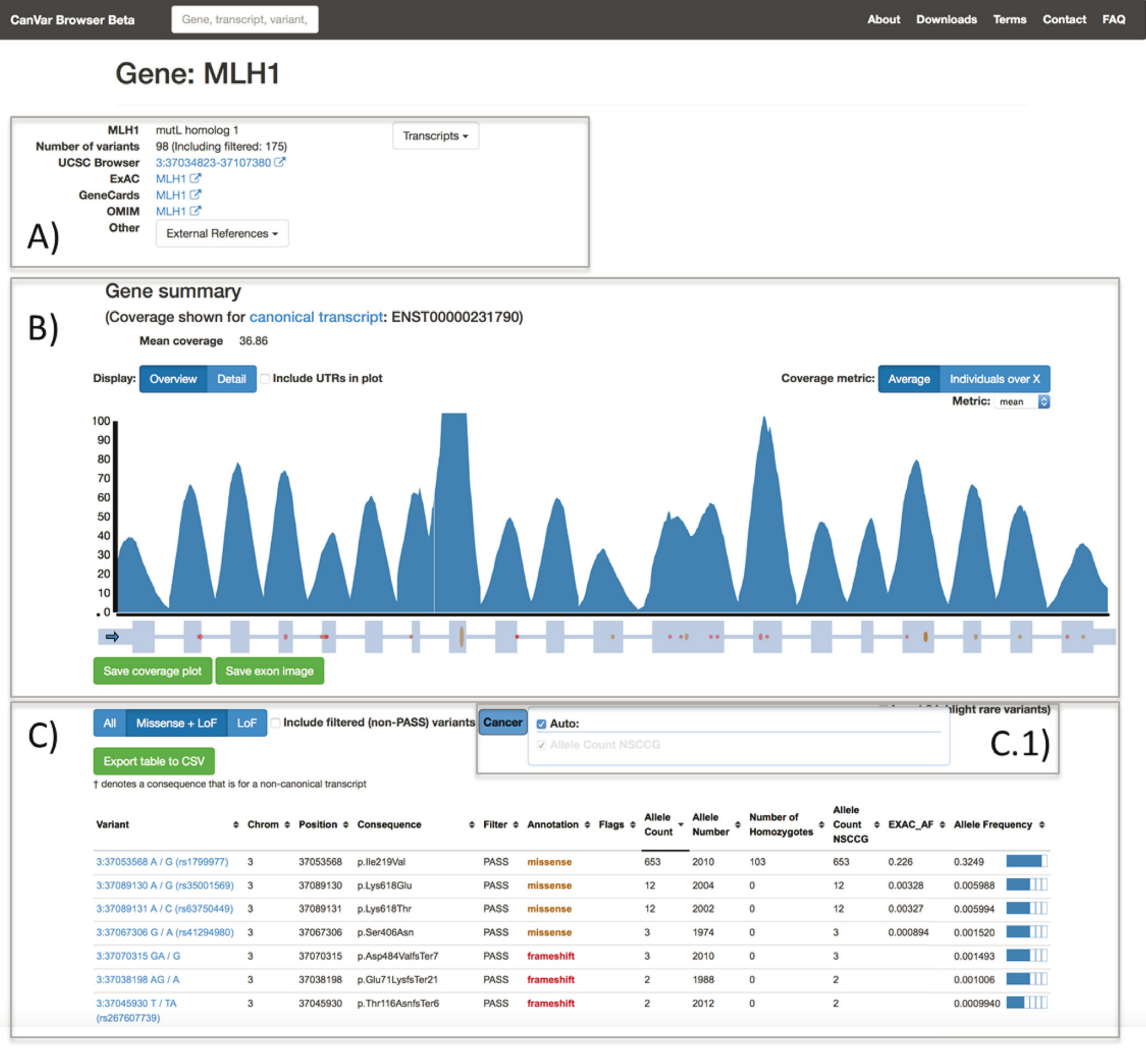
The Gene page is divided in to three parts. **A**) metadata and external links, including the ExAC page for a given gene; **B**) coverage plot and exon/intron structure **C**) table containing annotations and variant frequencies for each variant identified within a gene. The ExAC_AF column refers to the frequency from non-TCGA ExAC. The variant table has a menu C.1) which is used to select which cancer frequencies are displayed. Currently only NSCCG CRC samples are available.

**Figure 3.**
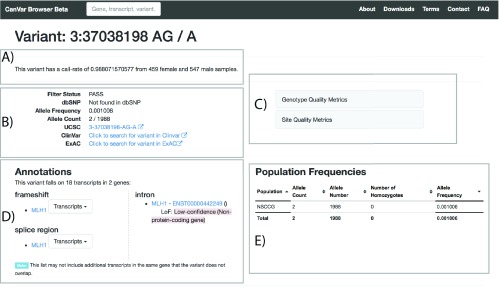
The Variant page can be divided in to five parts. **A**) Call rate of a given variant **B**) Metadata and external links, including equivalent ExAC page; **C**) Quality metrics **D**) Transcript annotations **E**) Frequency information in different studies.

CanVar utilises an adapted ExAC framework, providing SNV and INDEL frequency data and can be accessed via http://canvar.icr.ac.uk. The interface mirrors the ExAC browser available at http://exac.broadinstitute.org/ ^2^ and is divided in to three main parts: the front page ( Figure 1), the gene page ( Figure 2) and the variant page ( Figure 3).

### Front page

The front page ( Figure 1) contains a search bar where either genes or individuals variants can be queried. Genes are queried either by entering an HGNC gene name or ensemble gene ID. Individual transcripts within a gene can also be queried through entering an Ensembl transcript ID. Variants are queried either by dbSNP rsid or entering the chromosome, position, reference and alternate alleles. Additionally, whole regions can be queried, which opens a page similar to the gene view, providing coverage data and variants present in the queried region.

### Gene page

The Gene page ( Figure 2) first provides metadata and external links followed by a per base resolution coverage plot on top of the exon-intron structure of the gene of interest. These features default to the Ensembl canonical transcript but different transcripts can either be searched from the front page or selected from a drop down menu. A table provides frequency information and annotations for each variant identified within the gene assuming the worst effect in any transcript. The quality of a variant in the gene table is assessed by its filter status, obtained from the variant recalibration step of the GATK pipeline (Methods). To simplify the table display, users can select the cancers of interest. Non-TCGA ExAC frequencies are also displayed for each variant. Selecting a variant will open up the appropriate variant page.

### Variant page

More detailed quality and frequency information is provided in the variant page ( Figure 3). Links are provided to external resources such as the equivalent ExAC page and users can explore genotype, depth and site quality metrics. The call rate of each variant according to the QC thresholds (Methods) is provided at the top of the page. Care should be taken when interpreting variants with lower call-rates as they are typically more likely to be false positives. Annotation particular to different transcript can be browsed along with an assessment of loss of function variant quality according to the Loss-Of-Function Transcript Effect Estimator (LOFTEE - https://github.com/konradjk/loftee). The frequency of the variant across studies included within CanVar is also provided in a sortable table.

## Discussion

ExAC, the most comprehensive attempt at a large-scale aggregation of sequencing data, has been a great success, proving the usefulness of providing open-access population level genetic data for the research community. Here we present an adaptation of the ExAC framework to create CanVar, a cancer specific online resource for germline sequencing data.

CanVar currently provides SNV and INDEL frequency data, with associated annotations. As ExAC introduce new features it is anticipated that these will be merged in to future versions of CanVar.

The data currently catalogued in CanVar will provide a valuable resource for researchers investigating genetic predisposition to colorectal cancer and those engaged in delivery of clinical cancer genetic testing programs. It is expected that the utility of CanVar will increase as additional sequencing data is integrated through a number of different mechanisms: firstly, in-house sequencing of ongoing projects at the Institute of Cancer Research; secondly, applications for publically available data e.g. samples deposited in the Ensembl EGA archive and dbGap; and thirdly, collaborations with others engaged in the germline sequencing of cancer patients.

Only when the community fully embraces a policy of data sharing will resources such as ExAC and CanVar fulfil their potential. We therefore encourage all researchers engaged in cancer germline sequencing projects to consider sharing their data (email canvar@icr.ac.uk). Where consent or other factors preclude the sharing of the individual level data, we encourage others to adopt the ExAC framework to make their data available. To facilitate this we have made our adapted ExAC code available.

## Methods

## Implementation

### ExAC framework

CanVar is built upon the Python-based framework designed to accommodate the ExAC database downloaded from https://github.com/konradjk/exac_browser. A full description of the framework’s construction and optimisation is available from the ExAC browser publication ^2^.

Briefly, custom python scripts parse input data into a mongoDB database. These data consist of variant calls with VEP annotations (from VCF files) and sample coverage metrics (derived from BAM files) in addition to other annotation data in the form of downloaded flat files from dbSNP (for rsids), Gencode v19 (for transcript and gene structure), dbNSFP (for gene names and aliases) and OMIM (to link to the relevant OMIM entry).

The python Flask framework is then used to serve variant frequencies and associated annotations from mongoDB to webpages based upon HTML templates.

Hardcoded paths contained within the original code were altered and additional changes were made to the provided HTML templates to remove ExAC specific references and to make specific changes in the interface. For example, the gene results page was altered to annotate CanVar frequencies with ExAC frequency data and to allow for multiple studies to be viewed on the same table.

Full installation instructions with all software dependencies are provided at https://github.com/danchubb/CanVar/blob/master/readme.txt. The required python modules, installed using the pip package management system are described in https://github.com/danchubb/CanVar/blob/master/requirements.txt.

### Hardware

CanVar runs on a Dell PowerEdge R310 with 1x Intel i3-540 CPU and 4 GB DDR3 RAM using Apache version 2.4.6. The variant and associated annotation mongoDB files are 55GB in size.

### Website

The CanVar website itself can be accessed using any modern internet browser.

### Curation of colorectal cancer exome data within CanVar

CanVar currently contains summary level exome sequencing data from 1,006 early-onset familial CRC cases ^4^ from the National Study of Colorectal Cancer Genetics (NSCCG) ^5^. All samples had previously undergone quality control, ensuring the removal of those with: non-northern European ancestry, high levels of heterozygosity, sex discrepancy, poor call rate and contamination. The full sequencing and analysis pipeline is described in detail in the dataset’s publication ^4^. Briefly: all samples underwent exome capture utilising llumina’s Truseq 62 Mb expanded exome enrichment kit followed by sequencing using Illumina Hi-seq 2500 technology. Alignment to build 37 (hg19) of the human reference genome was performed using Stampy(v1.0.17) ^6^ and BWA(v0.5.9) ^7^ software. Alignments were processed using the Genome Analysis Tool Kit (GATKv3) pipeline according to best practices ^8, 9^. Analysis was restricted to capture regions defined in the Truseq 62Mb bed file plus 100bp padding. Combined individual level VCF files generated using the GATK 3 pipeline were assessed using variant quality recalibration (VQSR). In this step a variant is assigned a tranche which represents the sensitivity threshold required to call a given variant, the higher the tranche, the less confidence is given to a call. Variants are assigned a PASS value if they fall below the 99.0 tranche for SNVs and the 95.0 tranche for indels. Above these values, the sensitivity required for a given variant is reported in increments of 0.1 to provide users with the most accurate assessment of variant quality. The CRC cases were jointly called and subjected to VQSR alongside a larger set of exomes therefore calls may differ from those reported in previous publications. Finally, each variant was annotated using the Ensembl Variant Effect Predictor(VEP v78) ^10^ before being converted to the summary level site format required by the ExAC framework using custom python scripts.

### Data conversion to ExAC format

The ExAC framework requires individual level variant and coverage files to be converted into specific summary formats before they can be parsed into mongoDB.

***Variant frequency and annotation.*** Individual level vcf files are converted into a summary site format, providing allele count and frequency data for different groups in addition to depth and genotype quality data. For ExAC these groups correspond to ethnic groups whereas CanVar utilises this facility to instead group samples in to separate phenotypic classes, allowing the expansion of the database to contain data from a variety of malignancies. This process is accomplished using a custom python script https://github.com/danchubb/CanVar/blob/master/vcf_to_site_canvar.py which takes as input a VCF file and a list of which populations (or phenotypes) each contained sample belongs to. Variant frequencies and VEP annotations are then output according to QC parameters. In order to provide maximum sensitivity for users, minimum variant QC is imposed: requiring a site to be called in > 50% of samples and for an individual sample call to have a depth of > 2 reads with a GQ>20. All female Y chromosome calls are removed, as are male heterozygous Y and X calls.

***Coverage data.*** Per base coverage files are generated for each sample using the GATK DepthOfCoverage command. Individuals coverage files are then indexed using the tabix tool and average coverage across all captured bases is calculated across all samples using a custom python script: https://github.com/danchubb/CanVar/blob/master/average_coverage_calculate.py.

#### Data and software availability

The CanVar website is available at: https://canvar.icr.ac.uk

Latest source code: https://github.com/danchubb/CanVar

Archived source code as at the time of publication: 10.5281/zenodo.168019 ^11^

License: The source code is licensed using the same MIT open source license as ExAC ( https://github.com/danchubb/CanVar/blob/master/LICENSE).

##### Raw data

Raw alignment (BAM files) data on the 1,006 CRC samples have been deposited at the European Genome-phenome Archive with accession number EGAS00001001666. The availability of individual level data for future datasets included within CanVar will be specific to each study.
