# Supplementary material for: CanVar: A resource for sharing germline variation in cancer patients
Source: F1000Res. 2016 Dec 5;5:2813. [Version 1] doi: 10.12688/f1000research.10058.1 (PMC5200944; doi:10.12688/f1000research.10058.1)
Supplement: Peer review history

# Peer review: Referee response for version 1

**DOI:** 10.5256/f1000research.10838.r18681

**Published:** 2016-12-21

**Authors:** Pavel Vodička

**Affiliations:** 1 Department of the Molecular Biology of Cancer, Institute of Experimental Medicine, Academy of Sciences of the Czech Republic, Prague, Czech Republic

The study of Chubb *et al. *presents an online database and software tool Cancer Variation Resource (CanVar), developed on the basis of Exome Aggregation Consortium (ExAc) framework (sequenced exomes of cancer patients). The main aim of the database is to enable an open access to germline variant frequency. CanVar focuses on colorectal cancer, as it summarizes exome sequencing data from more than 1000 familial early-onset patients with this disease. Strikingly, the CanVar database catalogues almost 1.1 million variants and more than 100000 insertions/deletions. An additional advantage for the user are the data on associated annotations of variants and insertions/deletions.

 The information, which may be acquired from on the basis of the published database, may provide valuable information on gene variants and indels in populations assuming disease-specific context. The data that could be mined with the help of the present database may also find utilization in clinics, particularly in the context with mutational screening in cancer, which becomes to be routine.

 A sentence in Introduction (When undertaken by multiple agencies…) would benefit from re-phrasing into a more reader-friendly form.

I have read this submission. I believe that I have an appropriate level of expertise to confirm that it is of an acceptable scientific standard.

# Peer review: Referee response for version 1

**DOI:** 10.5256/f1000research.10838.r18219

**Published:** 2016-12-20

**Authors:** Laura Valle, Victor Moreno

**Affiliations:** 1 Hereditary Cancer Program, Catalan Institute of Oncology, IDIBELL, L'Hospitalet de Llobregat, Barcelona, Spain; 2 Unit of Biomarkers and Susceptibility, Catalan Institute of Oncology, IDIBELL and CIBERESP, Hospitalet de Llobregat, Barcelona, Spain; 3 Department of Clinical Sciences, School of Medicine, University of Barcelona, Hospitalet de Llobregat, Barcelona, Spain

In this article Chubb *et al.* ^1^ describe a software tool (CanVar) that the authors have developed to make publically available germline variant frequency information from sequenced exomes of cancer patients. Most importantly, they have used the Exome Aggregation Consortium (ExAc) framework, a tool the scientific community is currently very familiar to, in order to facilitate its access and use. The incalculable value of the open accessibility to the germline variation data obtained from >60,000 exomes provided by the Exome Aggregation Consortium (ExAc) seem to finally begin to reach disease-specific cohorts, as it is the case of CanVar. Hopefully this will soon become a reality not only for cancer but also for other common diseases. This information, together with the variation frequencies observed in the general population, is key when trying to evaluate the pathogenic relevance of disease-predisposing genes and/or variants, not only for novel candidate genes but also for well-known susceptibility genes.

 So far, the data available through CanVar correspond to the 1,006 exomes of early onset familial colorectal cancer cases recently studied by the same group ^2^. Being this a very insightful cohort in the field of colorectal cancer predisposition, much needs yet to be done to make CanVar a relevant routine tool for the scientific community, and it is the responsibility of all of us to make this possible. The tool is already available, so I encourage all researchers with germline exome sequencing data in cancer patients to submit their data to CanVar, as larger representation of tumor types, populations and patients in general is required. I also would like to encourage researchers to use these extremely useful data in their cancer predisposition studies and to increase the visibility of CanVar among their colleagues and peers.

 Despite the so far limited availability of germline exome sequencing data from cancer patients, a huge amount of data has been gathered in the last years from genome-wide association studies and exome SNP arrays. This information would be of value if added to CanVar, at least the variants included in exome arrays and rare exonic variants included in genotyping arrays. 

 Another issue that needs to be contemplated is the implementation of filters for ethnicities/studies, anticipating the inclusion of data from other groups. Alternatively, as occurs in ExAC, data could be itemized by ethnicity/geographic origin and study.

We have read this submission. We believe that we have an appropriate level of expertise to confirm that it is of an acceptable scientific standard.

## References

[1] ChubbDBroderickPDobbinsSEHoulstonRS: CanVar: A resource for sharing germline variation in cancer patients [version 1; referees: awaiting peer review]. *F1000Research*.2015;5(2813) : 10.12688/f1000research.10058.1 10.12688/f1000research.10058.1 PMC520094428105316

[2] ChubbDBroderickPDobbinsSEFramptonMKinnersleyBPenegarSPriceAMaYPSherborneALPallesCTimofeevaMNBishopDTDunlopMGTomlinsonIHoulstonRS: Rare disruptive mutations and their contribution to the heritable risk of colorectal cancer. *Nat Commun*.2016;7: 10.1038/ncomms11883 11883 10.1038/ncomms11883 27329137PMC4917884
